# Polymer Capsules with Hydrophobic Liquid Cores as Functional Nanocarriers

**DOI:** 10.3390/polym12091999

**Published:** 2020-09-02

**Authors:** Joanna Szafraniec-Szczęsny, Małgorzata Janik-Hazuka, Joanna Odrobińska, Szczepan Zapotoczny

**Affiliations:** 1 Department of Pharmaceutical Technology and Biopharmaceutics, Faculty of Pharmacy, Jagiellonian University Medical College, Medyczna 9, 30-688 Krakow, Poland; 2 Faculty of Chemistry, Jagiellonian University, Gronostajowa 2, 30-387 Krakow, Poland; janikm@chemia.uj.edu.pl (M.J.-H.); odrobinska@chemia.uj.edu.pl (J.O.)

**Keywords:** encapsulation, polymer nanocapsules, oil cores, drug delivery, nanoreactors

## Abstract

Recent developments in the fabrication of core-shell polymer nanocapsules, as well as their current and future applications, are reported here. Special attention is paid to the newly introduced surfactant-free fabrication method of aqueous dispersions of nanocapsules with hydrophobic liquid cores stabilized by amphiphilic copolymers. Various approaches to the efficient stabilization of such vehicles, tailoring their cores and shells for the fabrication of multifunctional, navigable nanocarriers and/or nanoreactors useful in various fields, are discussed. The emphasis is placed on biomedical applications of polymer nanocapsules, including the delivery of poorly soluble active compounds and contrast agents, as well as their use as theranostic platforms. Other methods of fabrication of polymer-based nanocapsules are briefly presented and compared in the context of their biomedical applications.

## 1. Introduction

The development of micro- and nanocontainers capable of carrying and releasing vulnerable substances has become one of the most important issues in food, cosmetic and pharmaceutical industries as well as in agriculture, marine and chemical industry [1,2,3,4,5,6]. Many works have been devoted to the protection of encapsulated molecules to enhance their stability, achieve a high efficiency of action and improve bioavailability. Thus, the controlled delivery and release of cargo from different types of micro- and nanocarriers is of considerable interest in applications such as the fabrication of self-healing materials, protection of antimicrobial or anti-corrosion agents, but mainly the preservation and delivery of nutrients, fragrances, drugs and sensing applications [7,8,9]. 

Considering that many substances of interest exhibit limited solubility in water, several attempts have been proposed to encapsulate such compounds, deliver them effectively, and release in a controlled manner at the site of action. Colloidal particles such as dendrimers, micelles, capsules, liposomes and polymersomes exhibit adequate size, high loading capacity, and sufficient stability which make them frequently used in the mentioned applications [10,11,12,13,14,15,16,17]. However, several aspects need to be considered during the design and development of such carriers. This includes their stability under manufacturing and storage conditions, the complexity of fabrication and purification steps, the possibility of scaling-up the process and its cost [18].

Oil-in-water nanoemulsions (O/W) exhibit a great ability to carry large quantities of hydrophobic substances in the dispersed phase and protect them from degradation. Such emulsions consist of oil droplets coated by thin polymer shells and suspended in an aqueous medium [19,20,21,22,23,24,25,26], so they can also be treated as dispersions of nanocapsules. They may serve as carriers of corrosion inhibitors, pesticides, and antimicrobial agents [27,28,29]. Moreover, they are suitable for food additives (e.g., milk derivatives, beverages), cosmetics (e.g., lotions, gels) and dietary supplements and drugs (e.g., vitamins, chemotherapeutics), enhancing the uptake of an active compound by a factor of even hundreds in comparison to the microemulsion systems. However, the thermodynamic instability of such nanosystems significantly affects their shelf life. To prevent destabilization, which is manifested by creaming, coalescence, flocculation, sedimentation and Ostwald ripening, they are mixed with emulsifiers, typically low molecular weight surfactants or more sophisticated functionalized macromolecules. 

In this work, we review the recent development of polymer nanocapsules, focusing on the amphiphilic graft copolymers and hydrophobically modified polysaccharides, stabilizing oil nanodroplets (oil-core nanocapsules), which can be used as carriers of lipophilic compounds in various applications. We present various ways of stabilizing such vehicles, tailoring both the cores and shells of the capsules towards the fabrication of multifunctional, navigable nanocarriers and/or nanoreactors. We also present the current applications of the nanocapsules as well as their future perspectives for entering the market.

## 2. Fabrication of Polymer Core-Shell Nanocapsules

Due to the variety of available monomers and the diversity of macromolecular architectures, polymers have been widely used in nanotechnology. Due to the self-assembly of macromolecules, they enable the formation of structurally well-defined materials with precisely tailored multiple functionalities, serving as versatile nanovehicles and nanotools. 

The self-assembly phenomena of amphiphiles related to the presence of a dynamic balance between associated and free molecules enables the formation of lamellar or vesicular systems, depending on the structure of building blocks. Such formed micelles, or liposomes made of phospholipids, serve as carriers for selected marketed pharmaceutics, such as doxorubicin (Doxil, Evacet^TM^), amphotericin B (Amphotec, Abelcet^TM^, Fungizone), morphine (DepoDur), fenofibrate (Tricor^®^), rapamycin and sirolimus (Rapamune^®^) [30]. They have also been introduced into cosmetics and dietary supplements. However, the instability and limited loading capacity, especially for lipophilic compounds, attracted attention to capsular systems with the core-shell structure. 

Polymer nanocapsules are vesicular objects in which the encapsulated compounds are typically confined in an inner cavity surrounded by the outer membrane. The cargo substance can be either dissolved or dispersed in the core material but can also be attached to the polymer shell. The capsules can be obtained using various methods, including dispersion polymerization, self-assembly and templating synthesis, in which the polymer coatings are deposited on solid or liquid cores using either in situ interfacial polymerization [31] or layer-by-layer adsorption (LbL) of polyelectrolytes [32]. The concept of LbL involves the formation of ultra-thin multilayer coatings on charged surfaces by alternately dipping the plates in the solutions of oppositely charged polyelectrolytes, separated by washing the excess of each polyelectrolyte [33]. The commonly used cationic polyelectrolytes are poly(allylamine hydrochloride) (PAH), polyethyleneimine (PEI), poly(diallyldimethylammonium chloride) (PDADMAC) and poly (l-lysine) (PLL), and among polyanions typically poly(sodium styrenesulfonate) (PSS), poly(vinyl sulfonate) (PVS), poly(sodium 2-acrylamide-2-methyl-1-propanesulfonate) (PAMPS) and poly (D-glutamic acid) (PGA) are used. The concept of the LbL technique was adapted for colloidal particles that serve as removable templates leading to the formation of the capsules with liquid cores [32]. Such a procedure enables the utilization of macromolecules of complex structure [34] varying in charge or glass transition [35] to reach the coatings and capsules of desired functionality [36,37]. 

The choice of the technique for producing nanocapsules should take into account the physicochemical properties of the carrying compound, its stability under the conditions of capsule formation, encapsulation efficiency and its release profile. As commercial applications also generate waste, the consumption of solvents, the complexity of the preparation procedure and the total costs should be considered as well. 

The templating synthesis is commonly used as it allows the preparation of capsules of finely tuned properties, various compositions and morphologies, modified release kinetics, and controlled size [31]. The use of solid colloidal templates (e.g., polystyrene, calcium carbonate, silica) led to the formation of monodisperse carriers of well-defined properties [38,39,40,41]. Despite many desirable features, the fabrication of capsules templated on hard colloidal cores is not free from limitations. One of the most important ones is the necessity of etching the core, which often requires the application of harsh reagents, which can result in damage or modifications to the shell, the destruction of sensitive cargo, and the uncomplete dissolution of the template, reducing the loading efficiency. Moreover, the multistep procedure of the capsules’ formation and purification is typically tedious. 

On the contrary, the use of “soft” templates and liquid droplets enables the facile and efficient encapsulation of hydrophobic cargo compounds, with precise control over the parameters of the capsules (size and shell thickness) [42]. The capsules can be obtained in several ways, including emulsification-coacervation, nanoprecipitation, emulsification-diffusion, double emulsification, polymer coating and the LbL deposition of oppositely charged polymers on oil templates [43,44]. They can be loaded in a one-step process, without an intermediate step of core removal and purification of the dispersed capsules. Such an approach was successfully applied to prepare emulsion-based carriers capable of protecting enzymes against degradation [45,46] or delivering active pharmaceutical ingredients such as carvedilol, meloxicam, doxorubicin, methotrexate [47,48,49,50]. After the encapsulation of fluorescent dyes or quantum dots, they may serve as imaging vesicles [51,52], while the incorporation of magnetic particles allows for the precise navigation of nanocapsules using the external magnetic field [53,54]. Polymer nanocapsules can also serve as carriers releasing the cargo upon a given stimulus and nanoreactors for the reactions requiring a confined environment [55,56,57]. Further functionalization leads to the formation of multifunctional carriers [58,59,60].

## 3. Stabilization of Oil Cores of Nanocapsules

Although oil-in-water nanoemulsions offer many advantageous features, they may be undermined due to their insufficient thermodynamic stability. Taking into account that the increase in the oil droplet size results in a decrease in the interfacial surface area, the destabilization of nanoemulsions may occur spontaneously. To limit the processes leading to the growth of the size of the droplets, the use of additives that stabilize oil droplets in polar media is necessary. 

**Figure 1:**
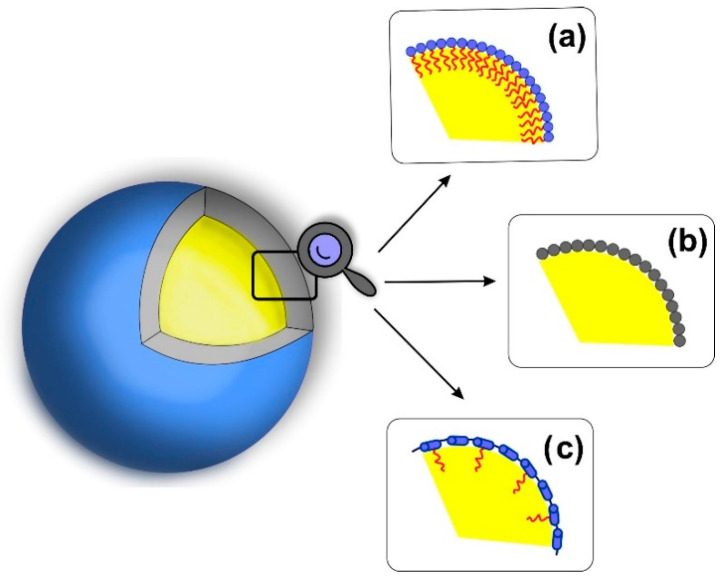
Different methods of stabilization of oil-in-water nanoemulsions using: (**a**) low molecular weight surfactants or block copolymers, (**b**) hard particles (Pickering emulsions), and (**c**) graft polymers.

The most commonly used stabilizers are low-molecular-weight surfactants [61]. The amphiphilic character of the molecules allows them to adsorb at the water–oil interface (Figure 1a). This reduces the surface tension, leads to breaking the drop surface continuity, and reduces its size. The use of charged molecules reduces their tendency towards aggregation due to the repulsive electrostatic interactions. However, a prerequisite for obtaining a stable structure is ensuring the appropriate concentration of surfactants. If the concentration is not sufficiently high, individual surfactant molecules can dissociate from the water/oil interface, and thus the uncontrolled release of the encapsulated compounds occurs. To ensure satisfactory stability of the emulsion systems, oil cores that are stabilized using ionic surfactants may also be additionally coated with multilayer polymer films using one of the LbL-based methods: (i) saturation, (ii) centrifugation and (iii) filtration [43]. In the saturation method, the amount of added polyelectrolytes is determined empirically to be sufficient to cover completely all the emulsion drops present in the system. The rinsing step occurring in the LbL technique applied for coating flat substrates is eliminated by avoiding the excess of polyelectrolyte. In the centrifugation method, the excess of polyelectrolyte added to the colloidal system is removed by centrifuging the suspension. The particles are then redispersed in a suitable medium. The process can be repeated many times until the free polyelectrolyte is entirely removed from the suspension. In the filtration method, the excess of polyelectrolyte is removed using filtration membranes that are permeable only to polyelectrolyte molecules. The removed volume of solution is continuously supplemented with a clean solvent to maintain a constant volume of the suspension, which minimizes the risk of aggregation. 

When preparing multilayer capsules templated on oil cores stabilized by charged low-molecular-weight surfactants, one should consider the nature of intermolecular interactions at the water/oil interface. Polymers interact with surfactants via electrostatic or hydrophobic interactions and hydrogen bonds. The selection of the appropriate surfactant-polyelectrolyte pair determines the formation of a stable interfacial complex, and thus stable capsules [62,63,64,65,66,67]. Such surface-active complexes exhibit tunable properties, which make them commonly used in industrial applications. 

An alternative to the application of low molecular weight surfactants is to use fine organic and inorganic particles, including silica, clay, hydroxyapatite, starch and cellulose, which accumulate at the interface of two immiscible phases and stabilize the droplets against coalescence [68] (Figure 1b). The mechanism of droplets’ stabilization relies on the formation of the steric barrier formed by the particles adsorbing irreversibly at the water-oil interface [69]. The lack of surfactants makes the Pickering emulsions exhibit higher stability, lower toxicity and a less complex structure [70,71].

Another approach is to use polymeric stabilizers, both in the form of homopolymers and block copolymers. The hydrophobic part of the molecule anchors firmly at the water/oil interface, while the hydrophilic part remains in the water phase and is strongly hydrated. While the use of homopolymers, e.g., partially hydrolyzed poly(vinyl alcohol), does not give completely satisfactory results, amphiphilic block copolymers, e.g., pluronics or synperonics based on poly(ethylene oxide) and poly(propylene oxide) work very well as emulsifiers [72,73]. Although these macromolecules are much less sensitive to changes in environmental conditions, significant dilution or changes in ionic strength can lead to their disassembly and, thus, to the destabilization of the emulsion. 

The use of amphiphilic graft polyelectrolytes for the stabilization of oil droplets has opened a new opportunity for designing more robust delivery systems [74,75] (Figure 1c). However, the field has not been widely investigated to date. The presence of a charged hydrophilic backbone and hydrophobic side chains makes them aggregate in an aqueous solution, forming hydrophobic pockets, and uncoil at the water–oil interface. Such polymers anchor the hydrophobic arms in the oil phase which results in better stabilization of the droplet than in the case of block copolymers or low-molecular-weight surfactants. Importantly, the capsules can be covered with ultrathin polymer films, which further increases their stability and allows tailoring for various applications.

## 4. Amphiphilic Polymers as Nanoemulsion Stabilizers: From Synthetic Graft Copolymers to Modified Polysaccharides of Natural Origin

**Figure 2:**
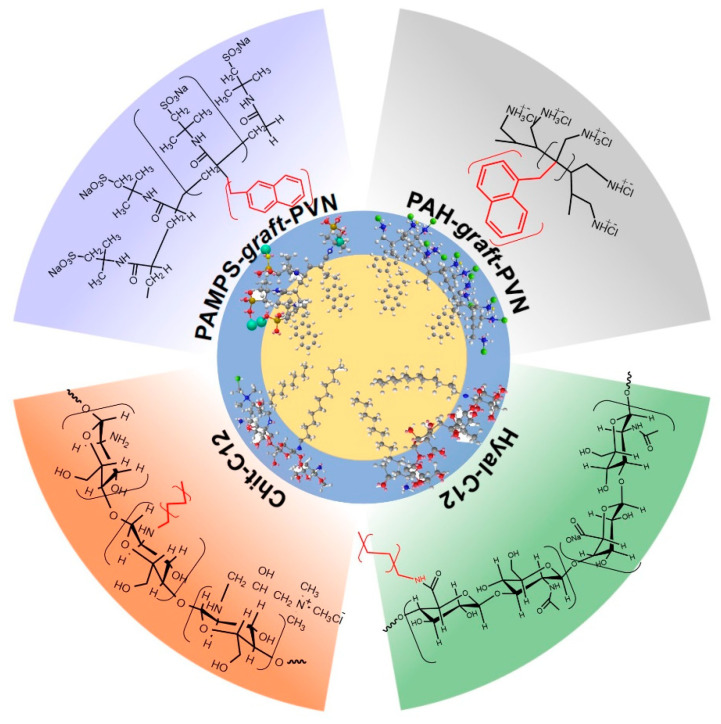
Nanocapsules stabilized by graft amphiphilic polymers and hydrophobically modified polysaccharides developed by Zapotoczny et al.

Zapotoczny and co-workers introduced a surfactant-free method of preparation of nanocapsules templated on the liquid hydrophobic cores [74] (Figure 2). The stabilization was provided by an amphiphilic graft copolymer, poly(sodium 2-acrylamido-2-methyl-1-propanesulfonate)-*graft*-polyvinylnaphthalene (PAMPS-*graft*-PVN). The dual chemical nature led to a spontaneous aggregation of the macromolecules in the polar medium and the formation of hydrophobic domains consisting of highly packed grafts, which can solubilize hydrophobic compounds. On the other hand, after the introduction of the toluene and emulsification, the polymer chain uncoiled partially at the water/toluene interphase, which enabled the stabilization of toluene droplets by the anchored polymer side chains. 

The authors hypothesized that the use of amphiphilic graft copolymer with a polyelectrolyte main chain ensures better stabilization of nanoemulsions than commonly used low-molecular-weight surfactants or block copolymers. This results from a higher number of hydrophobic grafts per macromolecule anchored at the toluene/water interface. To evaluate this hypothesis, nine series of polymers of various grafting densities and lengths of hydrophobic arms were synthesized using the nitroxide-mediated controlled radical polymerization (NMP) [76]. Thanks to the presence of ionic groups at the nanocapsule surface, further coating with thin polyelectrolyte-based films (using LbL approach) could be applied to enhance the stability of the obtained nanocarriers further.

The results revealed that the use of graft amphiphilic polyelectrolytes with a solvophilic backbone and solvophobic side chains might be useful for the preparation of spherical nanocapsules templated on liquid hydrophobic cores, which are suspended in water, without any additional stabilizers. The nanocapsules were found to be stable for at least 60 days after preparation. However, the stability of the nanocapsules depended on the composition of the used graft copolymers. In general, higher grafting density favored better electrostatically controlled stability of the nanocapsules dispersion, as expressed by higher absolute values of zeta potential. Further improvement in the nanoemulsion stability was obtained by coating them with thin multilayer films consisting of PDADMAC and PAMPS. The described strategy may allow for the formation of robust nanocarriers with a tunable size and composition, high loading capacity for lipophilic cargo, and long-term stability, serving as a nanodelivery system in various fields of applications, including biomedical and pharmaceutical ones.

A similar approach was described for amphiphilic poly(allylamine hydrochloride)-*graft*-poly(vinylnaphthalene) polyelectrolyte (PAH-*graft*-PVN), which was used as a stabilizer of toluene and *n*-octadecane nanodroplets [77]. The obtained nanocapsules served as efficient nanoreactors for photosensitized reactions, as presented for the photooxidation of perylene. The authors proved that molecular aggregates formed by PAH-*graft*-PVN in polar medium efficiently stabilized the sparingly water-soluble fluorescent probe and provided the nanoenvironment for the migration and transfer of the energy absorbed by the naphthalene units to perylene moieties. The efficiency of energy transfer increased after the introduction of the toluene phase into an aqueous polymer solution due to the formation of core-shell nanocapsules leading to attenuation of the interaction between the naphthalene groups. This led to the conclusion that the nanocapsules made of amphiphilic photoactive polyelectrolytes can serve as nanoreactors for numerous photosensitized reactions carried out in aqueous media. 

Given the growing interest in the application of colloidal particles in the biomedical field, the use of safe, biocompatible materials is highly demanded. Polysaccharides are natural polymers frequently used as components in many bionanotechnological applications. The presence of various types of functional groups in polysaccharide chains provides a variety of possible modification, including substitution with hydrophobic side chains, e.g., poly(alkylcyanoacrylate) or poly(methyl methacrylate), as well as short alkyl segments attached either via an amino or carboxyl group of the polysaccharide [78]. The self-assembly of amphiphilic polysaccharides in water leads to the formation of various formulations, depending on the content of hydrophobic groups covalently linked to the hydrophilic chain. Amphiphilic polysaccharides can form various structures, i.e., micelles, nanoparticles, microspheres and hydrogels that can carry both hydrophilic and hydrophobic compounds [79,80,81,82,83]. 

Chitosan (Chit) is one of the most commonly used polysaccharides. Its hydrophilic nature ensures good solubility in acidic media due to the protonation of amino groups. The presence of surface charge and the possibility of modifying the molecule with compounds with enduring electrostatic charge promotes the use of chitosan as a material for the construction of multilayer polymer films, coatings preserving cells during storage at low temperature, genes, proteins, drugs, and contrast agents carriers [84,85]. Numerous studies have also been carried out using modified chitosan to obtain injectable gels capable of transporting drugs. Chitosan also exhibits highly adhesive properties due to the interaction of cationic amino groups with anionic membrane proteins [86]. It has also been proven that chitosan possesses antibacterial properties [87]. All these features mean that the number of its biomedical applications is growing from year to year.

The use of the *N*-dodecyl derivative of cationically modified chitosan as a stabilizer of nanoemulsion was described by Szafraniec et al. [88] The surfactant-free method was based on ultrasound-assisted emulsification of an aqueous solution of hydrophobically modified chitosan with oleic acid, serving as the nanocapsule core. Spherical capsules of diameter varying between 250 and 350 nm were found to be stable in aqueous dispersion for at least 15 months of storage and resistant to the changes in the parameters of the external environment, including pH, ionic strength and dilution. The use of a charged polysaccharide derivative allowed the authors to deposit either cationically or anionically modified chitosan on the surface of the nanocapsules, tailoring their surface charge and improving their stability. The results of acute oral toxicity tests revealed that the capsules were non-toxic nanotransporters that opened up the opportunity of the application of Chit-based nanocapsules in the biomedical field.

Another important polysaccharide is hyaluronic acid (Hyal), a glycosaminoglycan (GAG) which is a linear copolymer of *d*-glucuronic acid and *N*-acetyl-d-glucosamine connected by β (1,3) and β (1,4)-glycosidic bonds. It is a highly hydrophilic anionic compound present in the form of sodium salt under the physiological conditions [89]. Due to high molar masses, reaching even 10^8^ g/mol, and strong intermolecular interactions in aqueous solutions, it can bind large amounts of water. It has been proven to be involved in many cellular processes, including cell migration and adhesion. In addition, high molar mass (>10^6^ g/mol) promotes anti-inflammatory and anti-angiogenic properties. Those properties made possible the successful introduction of this polysaccharide as a component of wound dressings, eye drops, and dietary supplements aimed at ensuring the proper functioning of articular cartilage [90,91].

A series of hydrophobically modified hyaluronate derivatives, differing in the degree of substitution and the length of hydrophobic units, were used for the stabilization of oleic acid droplets, obtained in the sono-assisted emulsification [88]. The results indicated that the proper balance between the degree of substitution of hydrophobic side chains and their length influence the size of the capsules. The carriers stabilized by hyaluronate modified with longer side chains (12 or 18 carbon atoms in the chain) were found to be more stable than those stabilized by C6- and C8-substituted polysaccharide (keeping similar degrees of substitution, c.a. 2%). An increase in grafting density led to the formation of capsules with the most favorable properties. The nanocapsules stabilized by the dodecyl derivative of hyaluronate (DS = 4.5%) were characterized by an average diameter below 180 nm, considered to be small enough for the efficient cellular uptake of nanocarriers [92]. The biodistribution studies indicated the preferential accumulation of hyaluronate-based nanocapsules, administered both orally and intravenously, in the liver and lungs. The capsules were taken up by liver sinusoidal endothelial cells and pulmonary microvasculature endothelial cells, as confirmed using in vitro tests. Thanks to the enhanced accumulation of nanocapsules in endotoxemic mice, they may serve as the liver- and lung-targeted delivery systems useful in the treatment of the organ injury linked to systemic inflammation. Importantly, the capsules were found to be non-toxic in the acute oral toxicity experiment, even when administered at doses equal to 2000 mg/kg b.w.

The influence of the molecular mass of Hyal on the properties and biodistribution of the capsules based on Hyal has not been systematically addressed, but it would be worth studying as the bioactivity of this polysaccharide may depend on it. For example, Kuehl and coworkers showed the dependence of the molecular mass of Hyal on its distribution in lungs, gastrointestinal tract, trachea and liver for several animal models [93]. The high molecular mass Hyal presents typically anti-inflammatory, immunosuppressive and antiangiogenic properties, promoting tissue integrity, while small polysaccharide fragments (below 100 kDa), are commonly inflammatory, angiogenic and immune-stimulatory. Hyaluronate oligosaccharides are also involved in the body’s alarm system [89,94,95]. Considering those properties, the therapies applying the Hyal-based capsules might be improved by careful selection of the molar mass of the polysaccharide.

## 5. Applications of Oil-Core Nanocapsules

The efficient delivery of lipophilic or hydrophobic active substances has been a challenging task for decades due to the possibility of their interaction with numerous components of human body fluids, the complexity of the uptake paths, and their limited solubility in aqueous media. Pharmacology is struggling with incomplete responses of treated cells or tissues, the inability to use drugs with high therapeutic potential, or the induction of severe side effects towards the healthy tissues. Thus, to overcome these drawbacks, several approaches have been developed which include loading the hydrophobic drugs in water-dispersible carries. They enable the delivery of sparingly water-soluble compounds into tissues or cells. This approach often uses the enhanced permeability and retention effect (EPR), passive targeting, [96] or some unique properties of the materials that enhance the tissue/cell penetration, e.g., the use of polysaccharide-based carriers may be treated as a potential source of energy by cells [97]. In addition to such non-targeted delivery systems, the carriers targeting given cells/tissues have been developed. The targeted therapy can be realized by decorating the carriers with appropriate ligands [98] or other materials with an affinity to specific cells/tissues [99], including stimuli-responsive polymers that are able to change their properties in response to a specific microenvironment [100].

### 5.1. Controlled Delivery and Release of Active Compounds

The EPR effect enables not only a prolonged circulatory half-life and improved biodistribution of some active compounds, but also their enhanced accumulation in tumor tissues [101,102]. Thus, this phenomenon is critical for drugs, prodrugs and their nanocarriers, affecting their accumulation in a body [103]. In healthy tissues, only low-molecular-weight drugs can extravasate out of blood vessels, while inflamed vessels in tumors are highly permeable, i.e., have fenestrated endothelium, open interendothelial junctions, defective lymphatic drainage system [104,105]. The extravasation is possible for the objects with sizes up to a few hundred nanometers [106], while smaller molecules are not (entirely) retained in tumors as they may return to the circulation by a diffusion [107].

Taking into consideration the sizes of NPs and the EPR effect, it has to be highlighted that it is still unclear what the perfect and/or limiting size of NPs is for different kinds of applications, and this is one of the main reasons for slowing down or even not taking on clinical trials [108]. The NPs with a hydrodynamic diameter of 100–400 nm are said to be optimal for passive tumor targeting. However, some reports claim a much better effect, e.g., for 30-nm large micelles than 100-nm ones [109], and the debate is still ongoing. More recently, it was reported that tumor vascular pores are not responsible for NPs’ extravasation, while the transport is realized actively by endothelial cells [110].

The numerous studies on polymer nanoparticles/nanocapsules claim their potential for passive targeting. Specifically, Szczęch and Szczepanowicz showed well-characterized polymeric core-shell nanocapsules to be vehicles capable of encapsulating various hydrophobic compounds for biomedical applications [111]. The multilayered capsules were obtained using the LbL method and further functionalized with polyethylene glycol (PEG) chains. The accumulation in the areas with permeable vascularity would be possible due to the prolonged circulation time and avoidance of binding with immune cells or proteins in the blood. Another group tested clinically approved Feraheme^®^, the iron oxide NPs which generate a signal in magnetic resonance imaging (MRI). Although analogous therapeutic NPs encapsulating docetaxel exhibited different properties, particularly in terms of size, the accuracy of co-localization was higher than 85% [112].

The other strategy used to deliver anticancer drugs is targeted therapy. This resulted from the specific features of the microenvironment surrounding tumor cells, i.e., low pH, a high level of glutathione, the presence of particular enzymes, and reactive oxygen species. Several studies have been directed towards altering the structure or properties under chemical, physical, or biological environmental changes, including pH, redox potential, enzymes’ activity, temperature, or light [113,114,115,116]. The response of stimuli-responsive polymers to a given stimulus may result in charge conversion or size changes [117]. This phenomenon is believed to improve targeting of the delivery systems, enhance tissue penetration or control release [118,119]. 

Surface charge is one of the crucial factors which influence cellular uptake, blood clearance, biodistribution, toxicity, etc., as it determines the interactions of NPs with biological systems. Positively charged NPs are often considered to be more toxic than the negatively charged ones, as they can easily enter cells and bind to negatively charged DNA. Moreover, positively charged DDSs are prone to opsonization that leads to the formation of the “protein crown” on their surface [120]. Charge-convertible polymers can change their charges due to certain environmental conditions or specific stimuli. Regarding the tumor targeting, the change in surface charge from negative or neutral into positive enhances targeting at a tumor site. However, such nanocarriers cannot have a positive charge permanently as they would exhibit toxicity for blood cells [121,122,123]. Another approach based on bio-orthogonal chemistry resulted in the preparation of a pH-responsive hydrazine network from poly(hydrazide) and functionalized dextran, which led to the fabrication of nanocapsules releasing a cargo in acidic conditions [124]. Nevertheless, it should be emphasized that while designing particular nanocarriers, one has to consider the properties of the whole system, including surface charge, size, chemical composition, shape, mechanical properties and shell thickness. 

### 5.2. Multifunctional Polymer Nanocapsules

Targeting delivery systems based on receptor recognition is an intensively studied topic in the context of therapy and diagnosis. Moreover, such structures are developing towards theranostic agents. The studies focus mainly on attaching ligands to the surface of a nanocarrier to enable selective binding to the extracellular matrix of diseased (mainly tumor) tissues or receptors overexpressed on cells or blood vessels. However, passive targeting of unmodified vehicles taking advantage of their specific surface properties is also of high interest.

There are plenty of ligand candidates for decorating of delivery systems, including small molecules, oligosaccharides, or even smaller saccharide entities, monoclonal antibodies, peptides, or aptamers [125,126]. Receptor-mediated delivery is realized through the endocytosis mechanism [127], while not all ligand-receptor pairs can internalize drug delivery systems (DDS). Those not internalized release a cargo near the outer cell surface, and transportation into the cell is run by passive diffusion or normal cell transport mechanisms [128]. 

**Figure 3:**
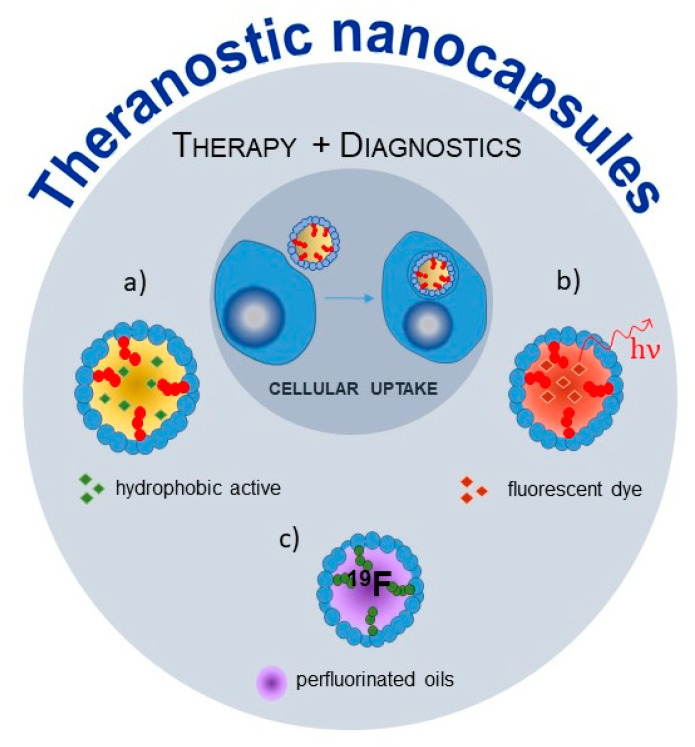
Functionalized nanocapsules for theranostic applications. The hydrophobically modified, polysaccharide-based nanocapsule encapsulating hydrophobic actives in oil core: (**a**) intracellular delivery of hydrophobic actives, (**b**) fluorescent dyes for imaging and capsules detection and (**c**) perfluorinated compounds for ^19^F NMR/MRI.

Nanoscale DDS, especially polysaccharide-based, may be decorated by various ligands. Among the most promising ligands that may be used for targeted therapy, biotin, folic acid, mannose, pullulan and antibodies can be distinguished [129,130,131,132]. Yang et al. described a vitamin-E-based nanoemulsion with promising properties as the theranostic platform. Using core-matched technology, the authors obtained carriers of the hydrophobic drug (paclitaxel) and hydrophilic imaging probe (sulforhodamine B) [133] (Figure 3).

In the case of smart targeting without extra ligand decoration, the most widely used pair of macromolecule and specific receptors is Hyal, which binds specifically to a few types of receptors, i.e., cluster of differentiation 44 (CD44), lymphatic vessel endothelial hyaluronan receptor-1 (LYVE-1), receptor for Hyal-mediated motility (RHAMM), hyaluronan receptor for endocytosis (HARE), among others. There is also a group of lymphocyte receptors, which bind specifically with sulfated polysaccharides, especially heparin sulfates, a ubiquitous and structurally diverse family of sulfated glycosaminoglycans [134]. The group of C-type lecithin receptors, most widespread in the immune system, recognizes carbohydrate structures. One of them, DC-SIGN receptor, is expressed by human platelets and megakaryocytes [135]. Thus, targeting directed towards these areas may be possible. Specifically, Hyal-based nanocapsules encapsulating oleic acid were examined as tumor-targeting carriers exhibiting the ability to be taken up by cancer cells, to release the cargo inside cells. Examined vesicles were taken up preferably by cancer cells in comparison with normal cell lines, which suggested specific interaction of hyaluronic acid with CD44 receptors overexpressed on the cancer cell line [136]. In vivo examination of these capsules allows us to observe their biodistribution in the liver and lung (see paragraph 4) [88]. The presence of Hyal-specific receptors on the LSEC surface and the presence of phagocytic Kupffer cells within the liver suggests targeting by Hyal, while capsules found in the pulmonary microvasculature do not necessarily relate to the Hyal-dependent cellular uptake (Figure 3). 

Several studies reported the enhanced uptake of polyelectrolyte-based vehicles not necessarily associated with the receptor-mediated pathway. Bazylińska et al. showed LbL-coated nanocapsules, with liquid cores, based on various polyelectrolyte (PE) pairs: DNA and Chit [137], dextran, and Chit, PSS and PDADMAC, functionalized additionally with PEG [138,139]. Comparing the uptake efficiency of nanocapsules based on synthetic polymers and natural polysaccharides by cancer and normal cells, a few observations were made: (i) the uptake by cancer cells was more efficient than by normal ones, (ii) the nanocapsules with polysaccharide shell (dextran/Chit) were more efficiently taken up by the studied cells than the nanocarriers with the synthetic PEs layers, (iii) a stronger antitumor effect of the encapsulated drug appeared for positively charged nanocapsules (i.e., with the outer Chit layer). The authors concluded that the sugar-based units are stable in the blood circulation and extracellular fluid. Moreover, polysaccharides may be a potential source of energy for cancer cells and play the role of “Trojan horse”, as tumor cells alter their metabolism to support their rapid proliferation and expansion, requiring more energy [97]. Polysaccharides which exhibit good bioadhesive properties, e.g., chitosan, may be especially good candidates for the shell layers. Chitosan was shown to be adsorbed and/or inserted into the membranes, most likely due to electrostatic and hydrophobic interactions [140]. Ventura et al. reported chitosan-based microspheres that exhibit enhanced permeation through membranes due to active and passive transport mechanisms, delivering gemcitabine, an antitumor agent [141]. 

### 5.3. In Vivo Imaging

Nanoscale carriers were also successfully introduced as imaging systems, such as nanoparticles containing contrast agents for magnetic resonance imaging (MRI) (e.g., gadolinium, or manganese oxide) [142] or dual-modal contrast agents for MRI and fluorescence imaging [143], as well as other techniques like photoacoustic, and Raman imaging. [144] For example, Wilk and coworkers reported oil-core nanocapsules encapsulating quantum dots as fluorescent markers [145]. A promising and straightforward approach was also developed for multimodal imaging systems, e.g., trimodal MRI-photoacoustic-Raman NPs imaging system [146].

A new approach with considerable potential in biomedical studies is to use the “second color” imaging using other atoms, such as ^13^C, ^23^Na, ^31^P, or ^19^F, in addition to ^1^H in MRI. With this respect, the nanodelivery systems with liquid hydrophobic cores play a particularly important role, as many contrast agents are hydrophobic or lipophilic. A very promising direction in MRI development is using ^19^F as the abovementioned “second color”. ^19^F MRI offers several advantages related to the properties of fluorine: 100% natural abundance and spin 1/2, a gyromagnetic ratio comparable with hydrogen and the lack of endogenous ^19^F [147]. Perfluorocarbons (PFCs) are molecules of special interest as they are rich in C-F bonds, but their application is not straightforward due to the significant hydrophobicity [147,148,149]. Among the most intensively studied PFCs, a few should be highlighted: perfluoroctyl bromide (PFOB), tetra(perfluorotertbutyl)pentaerythritol (PERFECTA) and perfluoro-15-crown-5-ether (PFCE). The last two are of particular interest due to their single-resonance, which enhances a signal in magnetic resonance [150]. Importantly, they are in a liquid state at physiological temperature. Thus, considering the in vivo applications of PFCs as contrast agents, they need to be formulated into delivery systems based on the oil-core structure. The conventional approach uses low-molecular-weight surfactants as emulsifiers. However, more advantageous for bioimaging but also challenging is the preparation of nanoemulsions without these additives [147]. The Hyal-based capsules were prepared for PFCs’ encapsulation. The Hyal macromolecules derivatized by attachment of perfluorinated alkyl chains were used for shell-building and stabilization of PFOB droplets dispersed in an aqueous medium [151] (Figure 3). Such formed nanocapsules were administrated to rats, and their localization in the aortas was detected ex vivo using a ^19^F NMR technique after only a single intragastric administration. This way, localization of the capsules with the same shell but different core carrying active compounds could be determined.

### 5.4. Magnetically Responsive Nanocapsules as Targeted Drug Delivery Systems and Chemical Reactors 

The delivery of bioactive molecules like enzymes, hormones, nucleic acids and transcription factors in a targeted manner remains a challenge in the development of novel therapies. In many cases, it is effective only in the cells where the therapeutic agent is delivered [152]. As human tissues are transparent to magnetic fields, the encapsulation of magnetic nanoparticles enables magnetic navigation with the external magnetic fields and accumulation of the carriers in the desired site of action [153,154,155] due to the increased microcapsule concentration at the magnetic site [156]. It meets the assumptions of theranostics, which combines therapy and diagnostics by the simultaneous delivery of pharmaceuticals and imaging agents within one entity [157,158,159]. 

Magnetic nanoparticles can be introduced into the polyelectrolyte capsules by either incorporation in the shell or encapsulation within the core. Several approaches have been made to prepare polyelectrolyte micro- and nanocapsules containing magnetic particles in the shell. The template-assisted process of their preparation included both solid and liquid cores. [160,161,162,163,164,165]. For hard colloidal particles, such containers can be formed in the process of pH-induced precipitation of iron salts in the capsule interior [166], followed by the coating by the appropriate core material [167,168,169,170]. However, due to a higher utility and favorable material characteristics, magnetic micro- and nanoemulsions have been proposed in recent years [171,172].

Magnetically responsive, liquid core capsules could be an attractive option for both drug delivery systems and cell therapy, including stem cells and immune cells used for regenerative medicine, and the treatment of genetic and cancer defects. Such systems allow for the magnetic guidance of living cells via uptake of microcapsules containing magnetic nanoparticles combined with the possibility of delivering bioactive molecules, either through sustained release or triggered release [173,174,175]. Podgórna et al. introduced the method in which the hydrophobic suspension of Fe_3_O_4_ nanoparticles was coated with a cationic polyelectrolyte layer with the aid of oil-soluble surfactant-docusate sodium salt [176]. A polyelectrolyte multilayer shell was formed using poly-l-lysine hydrobromide as a polycation and d-poly-l-glutamic acid sodium salt as a polyanion. Despite the high stability and easy preparation of such magnetically responsive polymer capsules, there are still a few drawbacks to overcome, such as the use of toluene/ethanol suspension of magnetic nanoparticles and the surfactants, which complicate the system and are not desirable in biomedical applications. As shown by Mu et al., the magnetic nanoparticles (oleic acid-modified magnetic nanoparticles) and drug molecules (dipyridamole) may be directly encapsulated into the interior of droplets without etching the templates and refilling it with the desired guest molecules, and also without using any low-molecular toxic surfactants [172]. The hybrid emulsion droplet cores were covered with biocompatible and biodegradable chitosan oligosaccharide and sodium alginate. It is worth mentioning that the method of capsules preparation was surfactant-free due to the self-emulsification of oleic acid in alkaline medium. Furthermore, the obtained multilayer shell of the capsules was pH-sensitive, which enables the controlled release of cargo molecules and magnetic navigation combined in one system. Despite the advantageous features, one drawback still may be pointed out. Despite the lack of low-molecular-weight surfactants, chloroform was introduced to the hybrid droplets to increase the solubility of the drug because of its poor solubility in oleic acid, which limits the biomedical application of such core capsules. 

Bae et al. described the formation of magneto-responsive nanocapsules containing oleic acid-stabilized iron oxide nanoparticles dispersed in various non-polar organic solvents, such as chloroform, dichloromethane and hexane [177]. The capsules were stabilized by block copolymer of poly(ethylene oxide) and poly(propylene oxide), i.e., Pluronic F127, which was chemically cross-linked with chitosan oligosaccharide. The obtained nanocapsules had the inner core with encapsulated magnetic nanoparticles, which was coated with a hydrophilic pluronic/chitosan polymer shell layer. The encapsulation did not affect the magnetization of the encapsulated iron oxide particles, which was concluded to be a high potential of the designed system to evolve into a magnetically-responsive nanoreservoir for water-insoluble anti-cancer drugs.

The use of graft copolymers as the stabilizers of decahydronaphthalene nanoemulsion was also suggested by Miao and co-workers [178]. The authors, however, described a more complex, ternary graft polymer, composed of poly(glycidyl methacrylate)-*graft*-[polystyrene-*ran*-(methoxy polyethylene glycol)-*ran*-poly(2-cinnamoyloxyethyl methacrylate)] (PGMA-*g*-(PS-*r*-MPEG-*r*-PCEMA)). The oil-filled nanocapsules loaded with superparamagnetic Fe_3_O_4_ nanoparticles were obtained after irradiation of the polymer with UV light that led to the cross-linking of the insoluble PCEMA chains, serving the capsules’ walls. The PS arms extended into the organic phase of the capsule core, and MPEG stretched into the water phase, providing the stabilization of decahydronaphthalene droplets. The authors confirmed that the capsules containing magnetite nanoparticles were superparamagnetic and susceptible to magnetic navigation.

Interestingly, cross-linked capsules maintained their structure after a series of magnetic capturing and shaking procedures, while the uncross-linked ones disintegrated after four cycles. Such behavior was concluded to be useful in tuning the structural stability and controlling the release of the cargo from nanocapsules using magnetic manipulations. The described capsular system can serve as an MRI contrast agent since the encapsulation of magnetic particles improves image contrast and prevents particle dilution. However, such an application requires the use of biocompatible, safe materials, which do not adversely affect the organisms. The simplicity of the synthesis would also be advantageous. Thus, many efforts have been made in the field of natural polymers, to modify them and use for the design of colloidal carriers of active compounds and diagnostic devices.

A simple, one-step method of encapsulation of magnetic emulsion without any additional organic solvents or low-molecular-weight surfactants was shown by Gumieniczek-Chłopek et al. [179]. The thermal decomposition method was applied to obtain magnetic nanoparticles consisting of wüstite cores of 6 nm in diameter and maghemite shells of 4.4 nm thickness. The formed superparamagnetic iron oxide nanoparticles (SPIONs) were then dispersed in oleic acid and emulsified with an aqueous solution of hydrophobically modified chitosan. The preparation process of such capsules involved only one step, i.e., ultrasound disintegration. The capsules with diameters on the level of 150 nm were stably suspended in water for 48 weeks of storage at 4 °C. Moreover, a high concentration of SPIONs in the oil phase (c.a. 100 g/L) ensured a strong magnetic response, which is necessary for their magnetically driven navigation. The obtained nanocapsules were also concluded to have a great potential for tailoring the magnetic properties and tuning the core-shell structure, which is desirable for the magnetically controlled delivery of active hydrophobic compounds.

**Figure 4:**
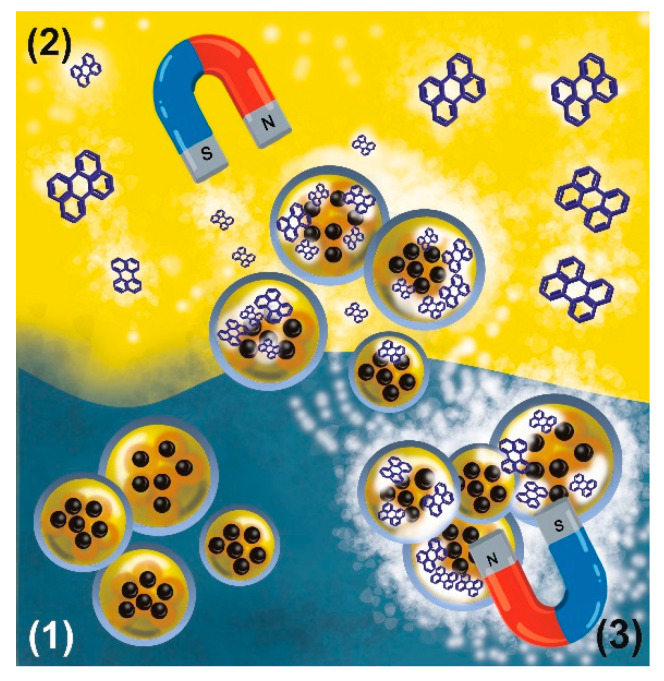
Magnetic navigation of the capsules with oil liquid core between two immiscible phases: (**1**) water with suspended nanocapsules filled with superparamagnetic iron oxide nanoparticles and (**2**) oil with dissolved perylene and loading process of “empty” cores with fluorescence probes; (**3**) drawing the loaded capsules into the aqueous phase.

The application of SPIONs-loaded nanocapsules was described by Odrobińska et al. [180]. Superparamagnetic iron oxide nanoparticles of 20–30 nm in diameter were coated with oleic acid and dispersed in the oil phase of chitosan-based core-shell nanocapsules. The nanocapsules were formed in the surfactant-free ultrasound-assisted emulsification of oleic acid or *n*-octadecane comprising hydrophobically coated SPIONs with an aqueous solution of the dodecyl-modified cationic derivative of chitosan. The obtained capsules (diameter of 500−600 nm) exhibited long-term stability (at least 2 months). The experiment on magnetic navigation revealed that SPION-loaded nanocapsules could be driven from the aqueous phase to the oil phase, where they were loaded with hydrophobic fluorescent probe, perylene, and transferred back to the polar phase without disintegration (Figure 4). Importantly, the nanovehicles served as reloadable photoreactors as shown for the photooxidation of perylene. Such a feature was also concluded to be very promising in the field of drug delivery.

The ability to transfer the nanovehicle between immiscible phases, e.g., water and oil, via magnetic navigation makes the abovementioned systems very promising in the field of magnetic oil/water separation in confined spaces, such as pipes, tubes and tanks [181]. Such reusable nanoreactors possess great potential for carrying out chemical reactions in a controlled and highly efficient manner. Such important features may be useful in environmental protection for the effective removal of toxic, organic pollutants from the wastewater [182,183]. Moreover, the confined environment of the nanovesicle, as well as its shape, microviscosity and polarity, can significantly affect the kinetics and thermodynamics of the reaction by creating transition states that lower the activation energy barrier [184]. Furthermore, carrying out chemical synthesis in a confined environment in comparison with processes taking place in bulk solutions allows for the improvement in reaction efficiency, more controlled and effective heat flow, and lower costs and material consumption of purifying reaction products [185]. 

The unique permeability properties of the capsule shells make these capsules excellent nano and micro reaction chambers for performing enzymatically catalyzed reactions [186,187,188,189]. The multilayer shells of capsules are semipermeable: permeable for small solutes; hence, the substrate and products of enzymatic reaction could permeate through the capsule wall while the enzymes are located inside and could be protected against high molecular weight inhibitors [189,190]. Most in vivo enzyme-catalyzed reactions occurring in a molecularly crowded environment and/or in a confined environment involve the controlled mixing of reactants and intermixing of the contents of the two or more compartments. That is why the triggered release of encapsulated molecules is a crucial feature. The externally triggered release has recently been shown to be possible by laser-light illumination [191,192,193,194,195], which causes changes in the permeability of the outer shell and even total disruption of the shell, finally resulting in the release of the encapsulated material, ultrasound stimulation [196,197,198,199], and via pH and temperature manipulation [200,201]. Moreover, the presence of ferromagnetic/superparamagnetic nanoparticles in a carrier shell may be utilized to achieve temporary increased membrane permeability [202]. These types of reaction chambers can act as valves for reagents, which can be closed on-demand as the external trigger stops. 

There are also many literature reports describing reactions in capsules in the hydrophilic/aqueous environment like the abovementioned enzymatic reactions. The fabrication of fluorescent rare-earth phosphates, reduction reaction or p-nitrophenol to p-aminophenol are just two examples [203,204]. However, there are very few literature reports describing the reactions in hydrophobic capsules’ cores. Odrobińska et al. described the photooxidation of the hydrophobic fluorescent probe–perylene in chitosan-based nanocapsules templated on liquid cores composed of oleic acid [180]. The presence of superparamagnetic iron oxide nanoparticles allowed for nanocapsules’ navigation in a magnetic field gradient and their transfer between the water and oil phase with dissolved perylene. This way, the capsules were loaded with a hydrophobic reactant, which was later photooxidized upon transferring the capsules to the aqueous phase. Such capsules may serve as novel, reusable and easily reloadable versatile reaction chambers for highly hydrophobic reactions in oil, confined environments and energy storage devices [205]. 

## 6. Summary

Recent developments in the fabrication and applications of colloidal polymer particles with a core-shell architecture were demonstrated. We described various ways of tailoring both cores and shells of the capsules towards the fabrication of multifunctional, navigable nanocarriers and/or nanoreactors. Particular emphasis was placed on emulsion-templated polymer nanocapsules as they provide efficient encapsulation and delivery of hydrophobic compounds, their protection against the harsh environment, and triggered release. We described various means of oil droplet stabilization and compared them with the newly developed technique of surfactant-free fabrication of nanocapsules employing graft copolymers and hydrophobically modified polysaccharides. In Table 1, a brief summary of the reported here oil-core capsules is presented.

The amphiphilic character of the above-mentioned polymers having a hydrophilic backbone and hydrophobic side chains enabled the formation of nanocapsules with oil cores stabilized by anchored grafts. The stability of the obtained nanocapsules depended on the hydrophobic/hydrophilic balance of the shell-forming polymer. Importantly, such polymers provided better stabilization than low-molecular-weight surfactants, which were further improved by the formation of multilayer coatings using the “layer by layer” technique of the absorption of oppositely charged polyelectrolytes.

We presented the applicability of multilayer emulsion-templated nanocapsules stabilized by either graft copolymers or hydrophobically modified hyaluronic acid and chitosan. The use of photoactive side chains was beneficial from the perspective of the formation of nanophotoreactors, whereas the use of hydrophobically modified polysaccharides opened a variety of biomedical applications, including liver-targeted delivery platforms. The encapsulation of superparamagnetic iron oxide nanoparticles within the oil cores led to the formation of magnetically navigated chemical reactors. The formation of nanocapsules with a shell composed of sodium hyaluronate modified with perfluorinated alkyl chains and the ^19^F-substituted oils as the capsules’ core allowed for the localization of the capsules administrated to rats, using an ^19^F NMR technique.

The development of functional polymer nanocarriers is a significant achievement in modern technologies, including medicine, biotechnology, chemistry and photoelectronics. They serve as versatile systems adaptable to various applications, such as drug, nutrients, pesticides and fragrance delivery, navigable (photo)reaction chambers, smart coatings and solar energy storage devices. Although many systems have already been intensely investigated, there is still a considerable area remaining for the development of better, tailor-made materials.

## Figures and Tables

**Table 1:** Brief summary with the most important properties of the oil-core nanocapsules.

Liquid Core	Shell	Additional Stabilizing Agent	Size	Application	Refs.
toluene	PAMPS-*graft*-PVN	-	50–300 nm	nanocontainers for hydrophobic fluorescent probes	[74]
toluene	PAH-*graft*-PVN	-	50–100 nm	nanoreactors for photosensitized reactions	[77]
oleic acid corn oil	Hyal-C12	-	100–350 nm	nanodelivery system (anticancer, anti-hypertensive), encapsulation of contrast agent (^19^F MRI)	[88,136,151]
oleic acid	Chit-C12	-	200–600 nm	reloadable, magnetically navigated nanoreactors	[75,179,180]
n-octadecane	Hyal-C12, Chit-C12	-	300–500 nm	imaging, morphology characterization	[75,88]
chloroform	silica	DTSACl ^a^	70 nm	encapsulation of a hydrophobic fluorescent probes	[25]
chloroform	PLL-PGA	AOT ^b^	100 nm	delivery of hydrophobic drugs, bioavailability improvement	[23,26]
linseed oil	PLL-PGA	lecithin	100 nm	drug delivery system for poorly water-soluble compounds, thus eliminating their potential toxic effects	[52]
chloroform	PLL-PGA, Fe_2_O_3_	AOT	140 nm	platform for multifunctional biomedical applications (controlled release of pharmaceuticals, hyperthermia treatment)	[160,165]
oleic acid	PSS, PLL, PDADMAC	C_12_(TAPAMS)_2_, ^c^ oleic acid	70–120 nm	pH-responsive and long sustained release nanocapsules	[57]
MT ^d^, MBT ^e^	PDADMAC	AOT	3–4 µm	emulsions of corrosion inhibitors	[27]

^a^ Dimethyloctadecyl[3-(trimethoxysilyl) propyl]ammonium chloride; ^b^ Docusate sodium salt; ^c^ N,N-bis [3,3′(trimethylammonio)-propyl] dodecanamide dimethylsulfate; ^d^ 2-methylbenzothiazole; ^e^ 2-mercaptobenzothiazole.
